# Off the Map: A Ten-Year Review of Coccidioidomycosis in Southeastern Michigan

**DOI:** 10.3390/jof12070518

**Published:** 2026-07-15

**Authors:** Daniel J. Muller, Carol A. Kauffman, Marisa H. Miceli

**Affiliations:** Division of Infectious Diseases, Department of Internal Medicine, University of Michigan Medical Center, Ann Arbor, MI 48109, USA

**Keywords:** coccidioidomycosis, *Coccidioides immitis*/*posadasii*, travel-associated infection

## Abstract

Recent studies suggest coccidioidomycosis can be found outside regions not considered to be endemic for *Coccidioides* species. We reviewed our experience with coccidioidomycosis at a large quaternary medical center in southeastern Michigan, an area not typically considered to be endemic for this infection. In the last decade, we cared for 18 patients with proven (11) or probable (7) coccidioidomycosis. All patients had a history of travel or prior residence in areas known to be endemic for *Coccidioides* species; for 16, the presumed source of exposure to *Coccidioides* was in southern Arizona. The median age was 64 (36–80) years. Thirteen patients had pulmonary coccidioidomycosis; manifestations included multiple lung nodules, consolidated pneumonia, diffuse reticulonodular infiltrates, chronic thick-walled cavitary lesions, and pleural involvement. Five patients had disseminated infection, including two with isolated coccidioidal meningitis, one with osteoarticular coccidioidomycosis, one with extra-thoracic lymphadenopathy in addition to diffuse lung infiltrates, and one with involvement of lung, mediastinal lymph nodes, and skin. Proven coccidioidomycosis was established by growth of *Coccidioides* species in culture in five patients, histopathological examination in five patients (one of whom also had a positive culture), and positive complement fixation (CF) test for *Coccidioides* antibody in cerebrospinal fluid for two patients. Probable coccidioidomycosis was documented for seven patients by positive titers for CF antibody to *Coccidioides* in serum. A careful travel and exposure history remains crucial for patients presenting outside *Coccidioides*-endemic regions, in which coccidioidomycosis may not be readily suspected and the diagnosis missed.

## 1. Introduction

Coccidioidomycosis is caused by *Coccidioides immitis*/*posadasii*, which exists in soil as a mold that produces conidia (sometimes called spores) and is seen primarily in the southwestern United States and Latin America [1]. After inhalation of arthroconidia that have developed in soil, the organism converts to the tissue form that is characterized by spherules/endospores. Although the organism occupies discrete environmental niches, the human host does not and may travel widely. Exposure to *Coccidioides* species can occur when people travel for work or vacation or when they move their residence. The disease may be indolent, presenting clinically after the person has moved from the area in which they were exposed to the organism. In this setting, low clinical suspicion may delay diagnosis and treatment. As early as the 1960s, reports from southern coastal areas and the upper Midwest commented on the risk of coccidioidomycosis to travelers and the difficulty of establishing a diagnosis in areas not known to be endemic for *Coccidioides* species [2,3,4,5,6,7,8,9]. Increased age has been recognized as a risk factor for coccidioidomycosis for several decades, most often in Arizona [10,11,12]. With an aging population, increasing numbers of retirees are traveling to the warmer winters of the southwestern U.S., and it is likely that more cases of coccidioidomycosis will be seen when these “snowbirds” return home to areas such as the upper midwestern states.

Since 1995, coccidioidomycosis has been a nationally notifiable disease, but only 28 states and the District of Columbia voluntarily submit surveillance data to the Centers for Disease Control and Prevention (CDC) regarding the disease [13]. In recent years, approximately 20,000 cases have been reported to the CDC yearly, but at least one report has estimated that the number of actual cases is 10–18 times higher than the number reported [14]. Recent surveys indicate not only that the number of cases from endemic areas is increasing, but also that more cases are being reported from areas outside of those traditionally established as endemic regions [12,15,16].

We wondered whether we were seeing an increase in the number of cases of coccidioidomycosis, what exposures these patients may have had to *Coccidioides,* and how the diagnosis was ascertained in this midwestern region not known to be endemic for *Coccidioides* species. We reviewed cases seen over the last decade at a large quaternary care center in southeastern Michigan.

## 2. Materials and Methods

### 2.1. Patients and Setting

This retrospective study was conducted at Michigan Medicine, a 1043-bed quaternary care hospital in southeastern Michigan. All patients 18 years of age and older who received either inpatient or outpatient care between 1 January 2015 and 1 March 2025 were screened for inclusion in the study. Patients’ records were screened if the terms “coccidioidomycosis”, “valley fever”, or “*Coccidioides* infection” were present. The study was approved by the University of Michigan Institutional Review Board; informed consent was waived due to the retrospective nature of this study.

### 2.2. Definitions

Proven and probable coccidioidomycosis were defined based on criteria modified from those of the European Organization for Research and Treatment of Cancer (EORTC) and the Mycoses Study Group Education and Research Consortium (MSGERC) consensus definitions for invasive fungal infections [17,18]. Proven coccidioidomycosis was defined when cultures yielded *Coccidioides* species, histopathologic examination or direct microscopy of a specimen revealed a distinctive tissue form of *Coccidioides*, or cerebrospinal fluid (CSF) tested positive for *Coccidioides* antibody or antigen. Probable coccidioidomycosis was defined when *Coccidioides* antigen was present in serum or urine or antibody to *Coccidioides* was present in serum in the setting of a compatible clinical presentation. Complement fixation (CF) and immunodiffusion (ID) antibody tests were performed by the Michigan Public Health Laboratory (Lansing, MI, USA). Enzyme immunoassay (EIA) for *Coccidioides* antigen was performed by MiraVista Laboratories (Indianapolis, IN, USA).

### 2.3. Data Collection

Data collected from the electronic medical record included patient demographics, underlying illnesses, including any type of immunocompromise, travel history, possible environmental exposure, type of infection (pulmonary versus disseminated), and diagnostic studies. All data were stored in a secure REDCap (Research Electronic Data Capture) database for further analysis [19].

## 3. Results

A total of 286 patient records were identified by the search terms. Only 18 patients met the definition of proven or probable coccidioidomycosis and were included in the study. There were equal numbers of men and women; the median age was 64 (36–80) years. Ten persons (56%) had underlying illnesses, including two (11%) who were immunocompromised (Table 1).

All 18 patients had their current permanent residence in southeastern Michigan, but all had acquired infection in areas known to be endemic for *Coccidioides* species. Sixteen patients (89%) had vacationed or previously lived in southern Arizona, one patient had driven through the San Joaquin Valley in California, and one patient had briefly worked in Tijuana, Mexico. Possible specific occupational or recreational exposure in endemic areas was documented for eight (44%) of the patients; four worked in construction, and four had engaged in outdoor activities, including hiking and landscaping. The number of cases of coccidioidomycosis that were seen yearly (1–3 cases) was constant during the 10-year study period.

Eleven patients (61%) had proven coccidioidomycosis; in nine patients this was confirmed by histopathology showing spherules and/or culture that yielded *Coccidioides* species, and in two patients by positive CF *Coccidioides* antibody in CSF. Seven patients (39%) had probable coccidioidomycosis based on serological results. Infection was confined to the thorax in 13 (72%) patients; disseminated coccidioidomycosis was present in 5 (28%) patients.

**Figure 1:**
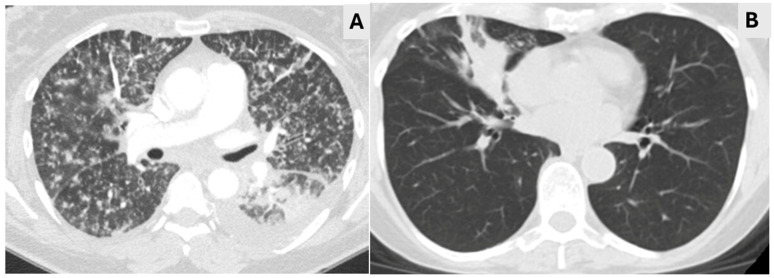
Acute pulmonary coccidioidomycosis. (**A**). CT of the thorax showing diffuse reticulonodular infiltrates in a woman who required intubation and mechanical ventilation. (**B**). CT of the thorax showing consolidated infiltrate in the right lower lobe.

**Figure 2:**
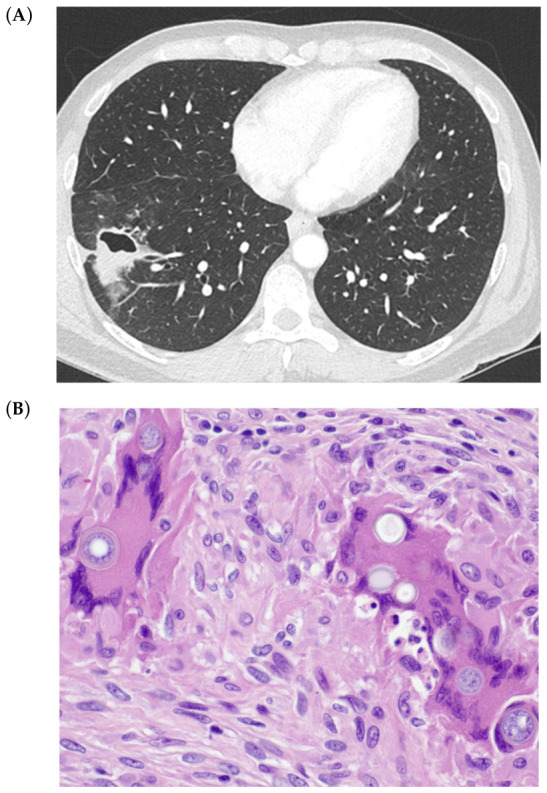

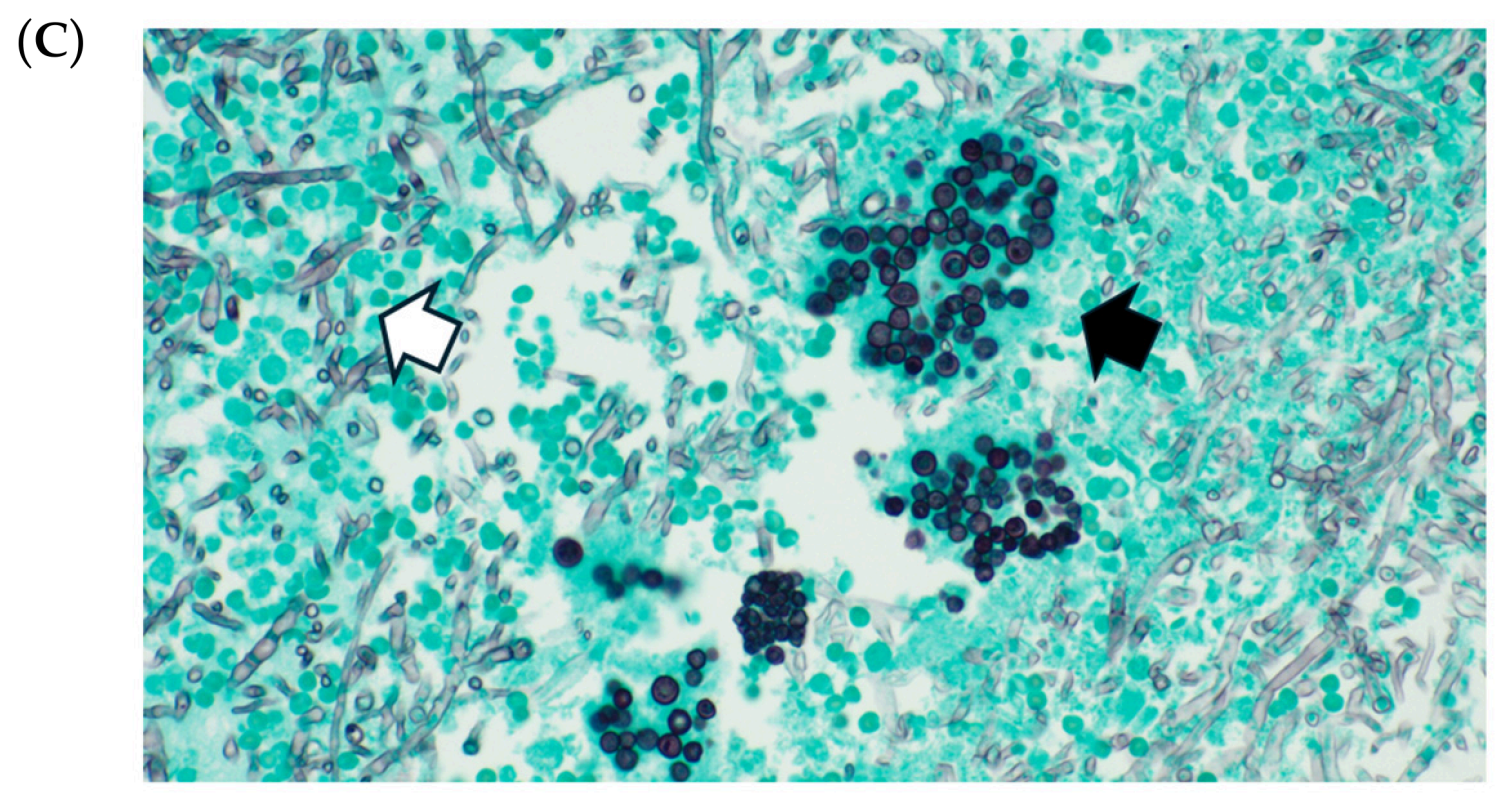
Chronic pulmonary coccidioidomycosis. (**A**). A large thick-walled cavity is noted on CT scan of the thorax in a patient with chronic cavitary pulmonary coccidioidomycosis. (**B**). Hematoxylin and eosin stain of lung tissue showing intact spherules of *Coccidioides species.* (**C**). Grocott methenamine silver stain of the same tissue highlighting endospores discharged from a spherule (dark arrow) and hyphae (light arrow) of *Coccidioides* species. The occurrence of hyphae within tissues is distinctly unusual and is likely related to the presence of the cavitary lung lesion.

Among the 13 patients who had pulmonary coccidioidomycosis, 6 were current or former smokers, and two had recently documented COVID-19 infection. Five patients were seen within 3–4 weeks of probable exposure with acute pulmonary coccidioidomycosis; CT findings showed diffuse reticulonodular infiltrates, consolidation, or ground-glass opacities (Figure 1). Eight patients had chronic disease that was manifested by relapsing symptoms with new infiltrates with or without cavity formation noted on CT imaging (Figure 2). Two patients had pleural involvement, one of whom manifested recurrent accumulation of large pleural effusions.

**Figure 3:**
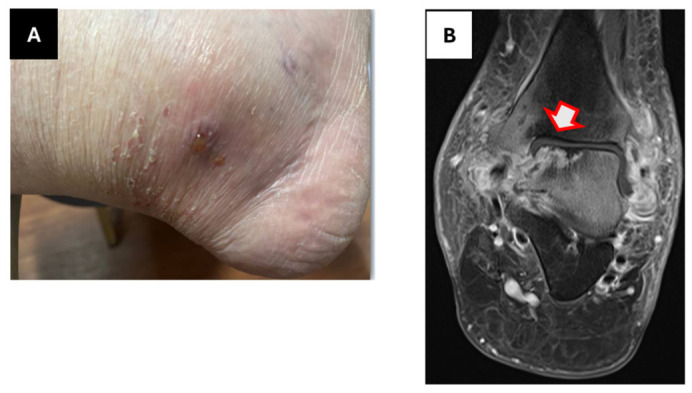
Osteoarticular coccidioidomycosis. (**A**). Swollen left ankle with punctate area from which purulent material had drained. (**B**). MRI of left ankle showing T1 hypointensity, marrow edema, and enhancement of the talus and adjacent distal tibia, and osseous destruction at the medial talar dome and posterior distal tibia (arrow).

Among the five patients who had disseminated coccidioidomycosis, one was a healthy woman with a miliary infiltrate on chest CT imaging and bilateral intra- and extra-thoracic lymphadenopathy; *Coccidioides* spherules were found on biopsy of a supraclavicular lymph node. Another patient who had received a kidney transplant 18 years prior and who was taking tacrolimus and 5 mg prednisone daily became ill several weeks after a trip to Arizona; lung biopsy yielded *Coccidioides* on culture and skin biopsy showed *Coccidioides* spherules. A patient with psoriatic arthritis who was not on immunosuppressive medication and who had a remote history of pulmonary coccidioidomycosis presented with septic arthritis/osteomyelitis of the left tibiotalar joint, which yielded *Coccidioides* on culture (Figure 3). The two patients with *Coccidioides* meningitis had the diagnosis established years before and remained on chronic fluconazole therapy with no relapse.

An initial clinical diagnosis other than coccidioidomycosis was made in seven (39%) patients; this included community-acquired bacterial or viral respiratory infection, pulmonary blastomycosis, or asthma exacerbation in six patients who had respiratory symptoms and signs. In one of these patients, histopathological demonstration of *Coccidioides* spherules with endospores led to both the correct diagnosis and confirmation of prior travel to Arizona. The patient with osteoarticular coccidioidomycosis was initially thought to have a flare of psoriatic arthritis; three weeks later, when a mold growing from synovial fluid was identified as *Coccidioides* species, the diagnosis was established.

The diagnosis of coccidioidomycosis was established by culture of lung tissue or bronchoalveolar lavage (BAL) fluid in four patients and synovium in one patient (Table 2). *Coccidioides* spherules were noted in biopsied tissue (lung, skin, lymph node, and pleura) in five patients, one of whom also had a culture that yielded *Coccidioides* species. Serological studies were available for 15 patients; the CF test was positive in 14 of the 15 (93%). The only negative result was noted in a kidney transplant recipient who had disseminated coccidioidomycosis. ID test results were reported for 12 patients, with positive results noted for 9 (75%). For six patients, the diagnosis of probable coccidioidomycosis was established by a CF titer > 1:2; three of these patients also had a positive ID test reported. For one patient with chronic relapsing pulmonary coccidioidomycosis, the only data available were reports from Arizona of positive CF titers. *Coccidioides* antigen was positive in urine for two of the four patients for whom this was ordered and in pleural fluid from another patient.

## 4. Discussion

We found that our experience with coccidioidomycosis in southeastern Michigan has remained relatively constant, with 1–3 new patients seeking care yearly over the last decade. All patients had a history of traveling or residing in well-described endemic areas for coccidioidomycosis. This contrasts with several reports that have raised concern for infection occurring in areas outside of those classically described as endemic for coccidioidomycosis [9,16,20,21,22,23]. Epidemiological studies have clearly shown specific locations in southeastern Washington and northeastern Utah in which acquisition of coccidioidomycosis has occurred [20,21]. In these same locations, *Coccidioides* species have been grown in culture or found by polymerase chain reaction techniques from soil samples [22,23]. Other studies raising concerns about whether coccidioidomycosis is occurring in diverse areas in the U.S. have relied on ICD/billing codes or public health surveillance data, which do not provide a history of travel/exposure to areas known to be endemic for *Coccidioides* species [9,16]. A review of medical records for our patients revealed one person for whom the initial notes did not describe travel history, and only when response to treatment for bacterial pneumonia was not achieved was a more complete history obtained. In another patient, an unexpected result demonstrating spherules in lung tissue led to a subsequent documentation of travel to Arizona.

The reference laboratory used by our medical center routinely performs CF and ID tests for *Coccidioides,* as well as tests for histoplasmosis and blastomycosis when “fungal serology panel” is requested. For several patients, a positive CF test was the initial finding that alerted clinicians to the possibility of coccidioidomycosis. Although the CF test is not typically recommended for the initial diagnosis of coccidioidomycosis because of its lower sensitivity than EIA tests for antibody [18], in our cohort we found it to be useful for alerting clinicians to consider coccidioidomycosis.

Most of our patients were older than 60 years, reflecting an increased risk for developing coccidioidomycosis with aging. An increase in the number of cases of coccidioidomycosis in older adults has been noted previously, and one study noted that recent migration to Arizona was an important risk factor in the development of the disease in older adults [10,11,12]. It is likely that the increasing movement of older adults from areas, such as the upper Midwest, into the southwest for both short-term winter trips and retirement contributes to an increased risk of developing coccidioidomycosis in older individuals.

The limitations of this study are several. We report on a small number of patients treated at a single medical center in southeastern Michigan, and our findings may not reflect those noted at other institutions. In addition, data were collected retrospectively, which carries the risk of introducing potential bias.

In conclusion, evaluation of our cohort from a large referral medical center serving southeastern Michigan suggests that coccidioidomycosis remains uncommon in this geographical area. All identified cases had exposure in areas that are known to be endemic for *Coccidioides* species, underscoring the importance of obtaining a thorough travel history to guide appropriate testing and timely diagnosis of coccidioidomycosis.

## Figures and Tables

**Table 1:** Demographics and underlying conditions in 18 patients with coccidioidomycosis.

Characteristic	Number (%)
Age–years (median, range)	64 (36–80)
Sex	
Men	9 (50%)
Women	9 (50%)
Race	
White	16 (89%)
Black	2 (11%)
Underlying illnesses	
Diabetes mellitus	5 (28%)
Chronic lung disease ^1^	3 (17%)
Rheumatologic disease	3 (17%)
Inflammatory bowel disease	2 (11%)
Solid organ transplant	1 (6%)
Congestive heart failure	1 (6%)
Cirrhosis	1 (6%)
Immunosuppressive medications ^2^	2 (11%)
None	8 (44%)

^1^ asthma (2), COPD (1); ^2^ tacrolimus (1), tumor necrosis factor antagonist (1).

**Table 2:** Diagnostic testing for 18 patients with coccidioidomycosis.

Type of Infection	Disease Type	Mycological Diagnostic Testing			
		Culture Positive	Histopathology Positive	Serology	
				CF	ID
Proven	Disseminated ^1^			1:4 (CSF)	NA
	Disseminated ^2^	Synovial fluid		1:64	Positive
	Disseminated ^1^			1:64 (CSF)	NA
	Disseminated		Lymph node	1:64	Positive
	Disseminated	Lung biopsy	Skin	Negative	NA
	Pulmonary		Pleura	1:32	Positive
	Pulmonary	BAL		1:32	Positive
	Pulmonary		Lung	NA	NA
	Pulmonary	BAL, sputum		NA	NA
	Pulmonary	BAL, sputum		1:16	Positive
	Pulmonary		Lung	1:16	Positive
Probable	Pulmonary			Positive ^3^	NA
	Pulmonary			1:16	Positive
	Pulmonary			1:16	Positive
	Pulmonary			1:2	Negative
	Pulmonary			1:2	Indeterminate
	Pulmonary			1:4	Negative
	Pulmonary			1:2	Positive

BAL: bronchoalveolar lavage; CF: complement fixation; ID: immunodiffusion; NA: not available; CSF: cerebrospinal fluid. ^1^ Central Nervous System infection. ^2^ Osteoarticular infection. ^3^ Reported positive, testing done locally in Arizona.

## Data Availability

Please add the corresponding content of this part.
